# Recommendations for strain elastography of the uterine cervix

**DOI:** 10.1007/s00404-024-07693-x

**Published:** 2024-08-29

**Authors:** Christine Rohr Thomsen, Maria Strandbo Schmidt Jensen, Pinar Bor, Mogens Hinge, Puk Sandager, Niels Uldbjerg

**Affiliations:** 1 Department of Obstetrics and Gynecology, Aarhus University Hospital, Palle Juul-Jensens, Boulevard 99, 8200 Aarhus N, Denmark; 2 Department of Obstetrics and Gynecology, Randers Regional Hospital, Randers NØ, Denmark; 3 Department of Clinical Medicine, Aarhus University, Aarhus, Denmark; 4 Center for Fetal Diagnostics, Department of Clinical Medicine, Aarhus University Hospital, Aarhus, Denmark; 5 Department of Biological and Chemical Engineering, Aarhus University, Aarhus N, Denmark

**Keywords:** Elasticity imaging techniques (E01.370.350.850.270), Ultrasonography (E01.370.350.850), Uterine cervical incompetence (C12.050.351.500.852.593.120), Induced labor (E04.520.252.968), Uterine cervix (A05.360.319.679.256)

## Abstract

**Purpose:**

Conventional vaginal strain ultrasound elastography, not based on shear-wave elastography imaging, can assess the biomechanical properties of the uterine cervix. This assessment may inform the risks of preterm birth and failed induction of labor. However, there is considerable variation in the approaches to strain elastography, including the placement of the region of interest (ROI). Therefore, our aim was to provide recommendations for cervical elastography.

**Methods:**

We conducted a literature review on (1) elastography principles, and (2) the cervical anatomy. Subsequently, we performed elastography scanning using a Voluson^™^ E10 Expert scanner with the BT18 software of (3) polyacrylamide hydrogel simulators, and (4) pregnant women.

**Results:**

Increasing the distance between the ROI and probe led to a decrease in the obtained strain value; a 53% decrease was observed at 17.5 mm. Similarly, an increased angle between the ROI and probe-centerline resulted in a 59% decrease for 40° angle. Interposition of soft tissue (e.g., cervical canal) between the ROI and the probe induced an artifact with values from the posterior lip being 54% lower than those from the anterior lip, even after adjusting for probe-ROI distance. Equipment and the recording conductance significantly influenced the results.

**Conclusion:**

Our findings inform recommendations for future studies on strain cervical elastography.

## What does this study adds to the clinical work

**Table:** 

The study presents recommendations for cervical elastography: the placement of the ROI, importance of distance, angle deviation, and default settings.	

## Introduction

The biomechanical strength of the uterine cervix plays a crucial role for the outcome of pregnancy. Thus, cervical insufficiency can cause second trimester abortion, while preterm cervical ripening may cause preterm birth [1–3]. In addition, the absence of cervical ripening at term is associated with post-term pregnancies and an increased risk of unsuccessful labor induction [4]. Currently, digital evaluation of the cervix remains the gold standard for assessing cervical consistency and, consequently, biomechanical strength. However, this method is hindered by low performance [5–8], highlighting the need for more reliable techniques.

One intriguing approach for assessment of cervical consistency involves transvaginal ultrasound combined with strain elastography [9–12], distinct from shear-wave elastography. Principally, elastography evaluates tissue deformation upon mild external compression of the tissue by the ultrasound probe [13] or internal compression, which can include pulsations from arteries among other factors [14]. After completing the recording, the operator places a Region Of Interest (ROI), and subsequently, the elastography software computes the strain values (deformation by force) of the ROI [15, 16]. Conventional ultrasound elastography can be based on changes in the B-mode image, whereas alternative approaches are based on Doppler techniques [17]. In addition to these ultra sound based methods, a number of mechanical devices have been developed but never implemented in the clinic [18].

However, several challenges must be addressed before optimal elastography results can be achieved. One challenge is controlling the pressure applied to the tissue during the elastography recording. This might be accomplished by incorporating a force-measuring system within the handle of the ultrasound transducer [19] or by using a device that applies repetitive predefined pressures. Another challenge is the absence of a natural reference material, like fat, close to the cervix. One approach to this challenge is to interpose a “ultrasound-friendly” synthetic reference material with known biomechanical properties between the ultrasound probe and the cervix [15, 20].

Other variables affecting elastography assessments are the distance between the ROI and the ultrasound probe, as well as the heterogeneity of the cervical tissue [16, 21–23]. Thus, recordings from the posterior cervical lip may be unreliable due to the interposition of the softer cervical mucus plug [20, 24] between the probe and the ROI when the scan is conducted with the probe in the anterior vaginal fornix. In addition, certain parts of the cervix such as the isthmic area dominated by smooth muscles, the mucus plug, the crypts along the cervical canal, the sub-epithelial area of the exocervix with glands, Nabothian cysts, and blood vessels, may be less important for the biomechanical strength of the cervix and should not be included in the ROI [25, 26].

Previous studies have addressed these challenges in various ways. Some have included the entire cervix in the elastography analysis, either by placing one ROI covering the full cervix [27, 28] or using multiple ROIs at different cervical locations, including the anterior and posterior cervical lip, the external and internal cervical os, and the cervical canal [21]. Others have focus solely on the anterior cervical lip [9, 10]. In addition, the approach to shaping the ROI also varies, with circular ROIs being common [9, 10, 21, 27], but square ROIs also described [11, 29, 30]. Moreover, the settings of the ultrasound system [9, 31] and the angle between the cervical canal and the probe-centerline can affect the obtained strain values.

**Fig. 1:**
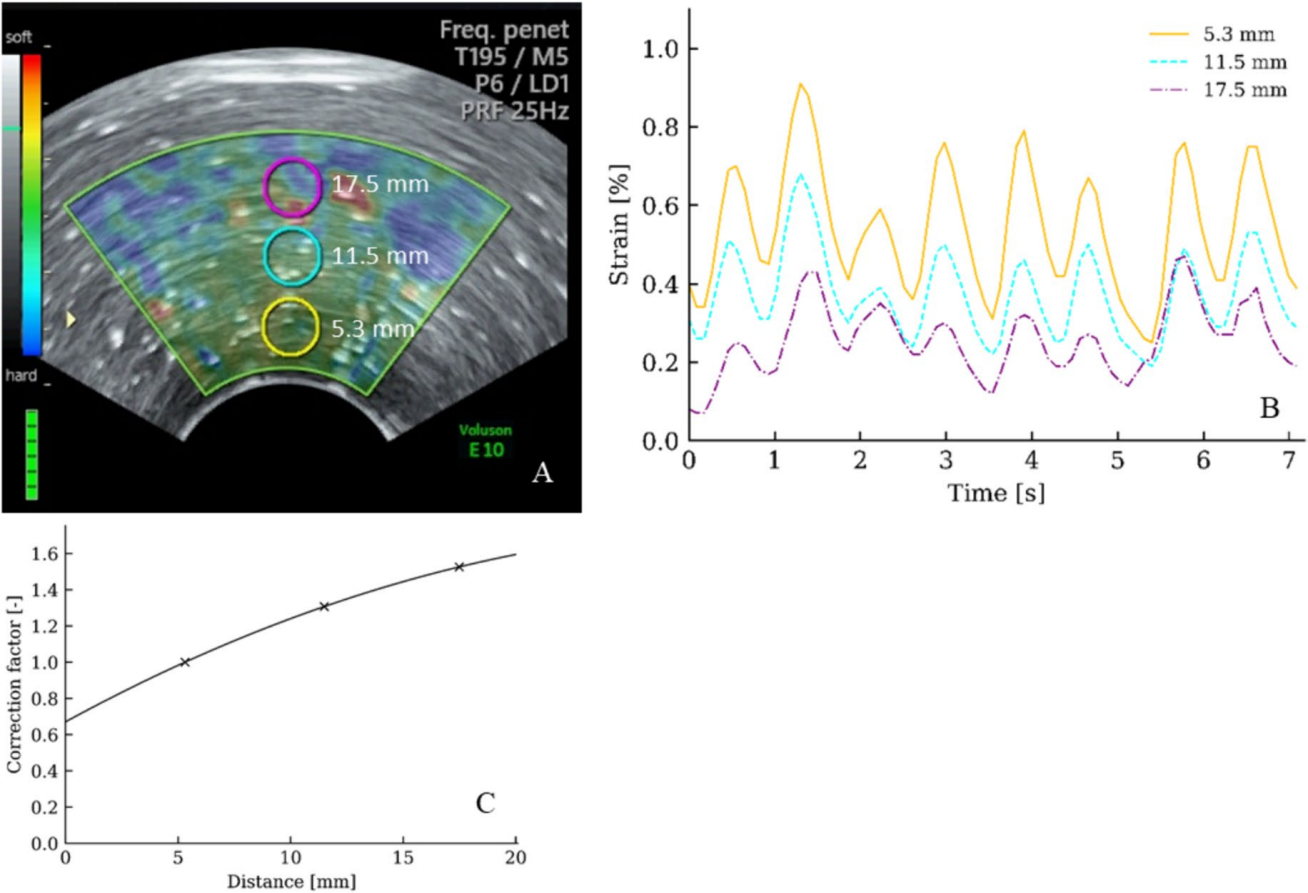
Distance to ROI **A** B-mode image of a simulator made of polyacrylamide hydrogel with the color box superimposed. Three ROIs (⌀ 5 mm) are placed with the center 5.3, 11.5 and 17.5 mm from the probe. **B** Strain curves obtained from the three ROIs when using roughly one compression per second. **C** The dots are based on ten recordings like that illustrated in **B**. Using polynomial regression, the correction function is given by *C* = 2.803⋅10^–3^ *x*^2^ + 2.675⋅10^–2^ *x* + 0.7737, where *C* is the amplified signal and x is the distance from the probe surface to the center of the ROI, measured in mm

**Fig. 2:**
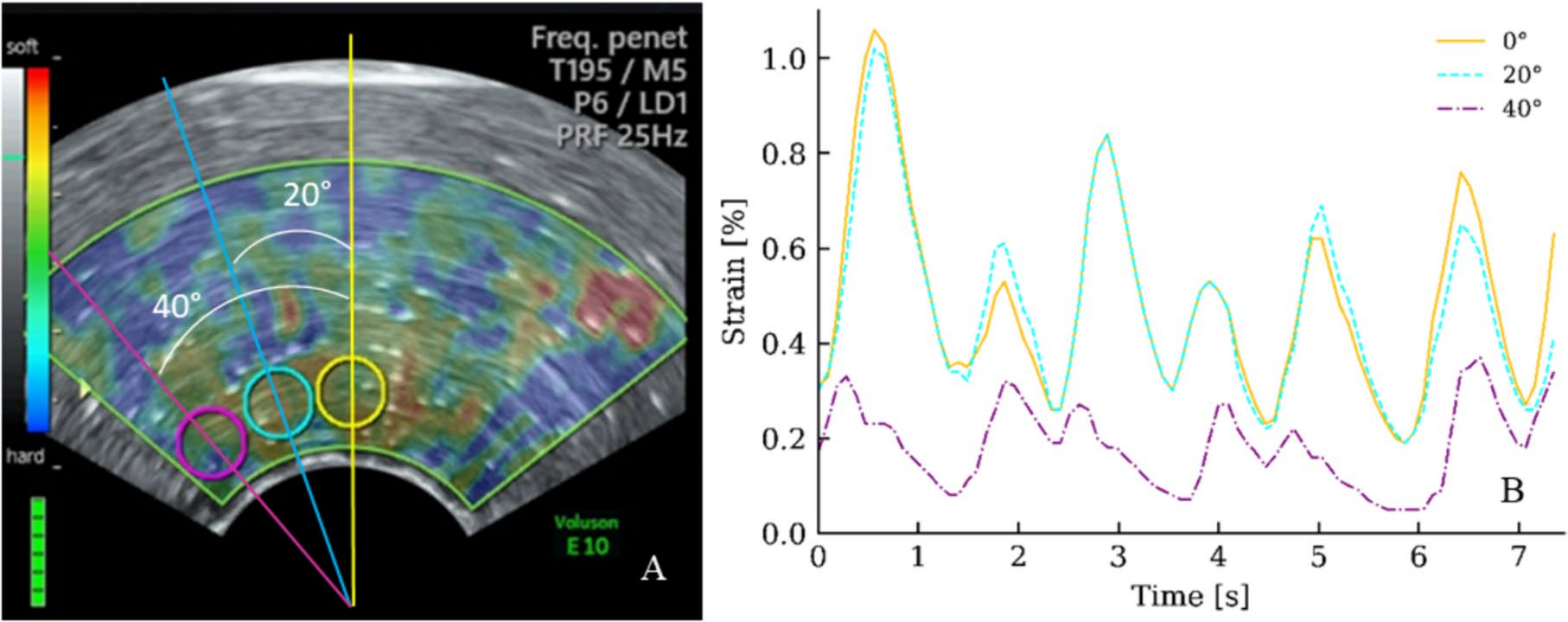
Angle from probe centerline. **A** B-mode image of a simulator made of polyacrylamide hydrogel with the color box superimposed. Three ROIs (⌀ 5 mm) are placed with the center at 0°, 20°, and 40° to the probe-centerline. To study 40° angle-deviations of the ROI, the angle of the color box has been increased from 75° (default) to 100°. **B** Displays the computed strain values obtained from the three ROIs

**Fig. 3:**
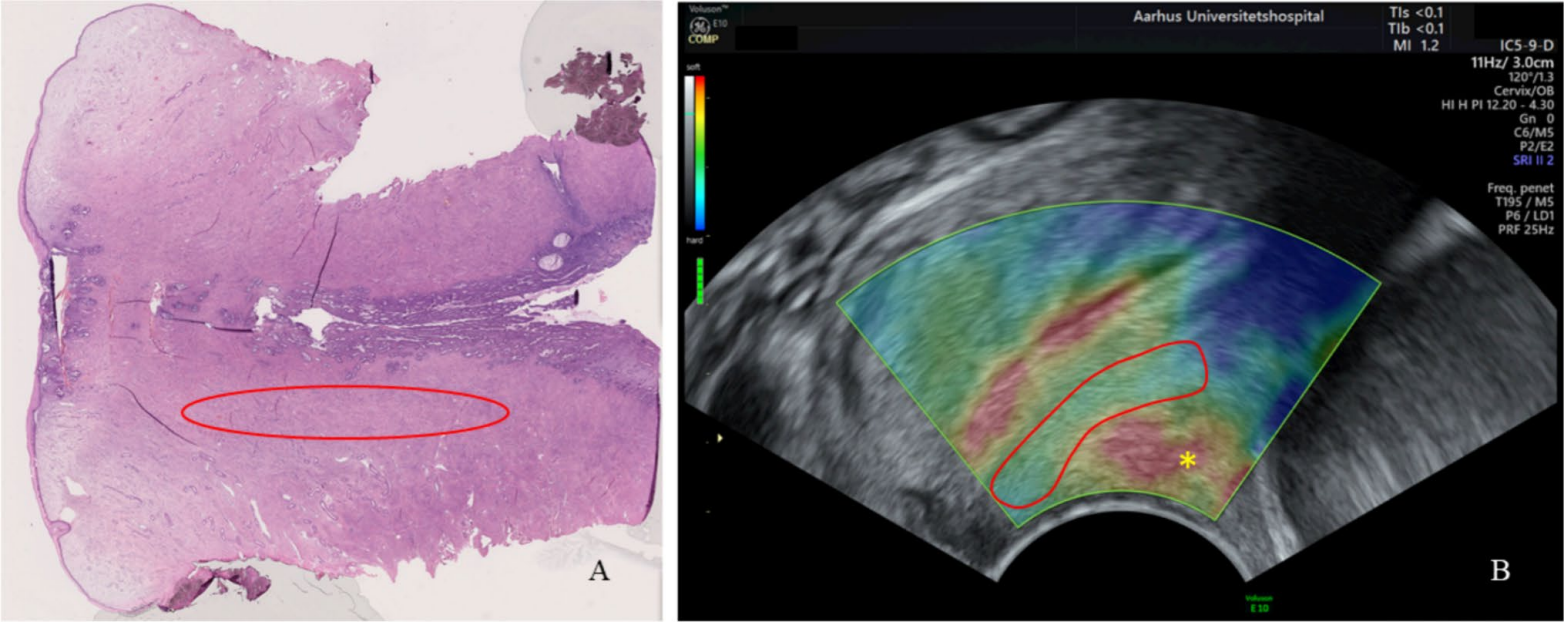
Anatomic area of interest. **A** Sagittal view of a hematoxylin and eosin (HE) stained uterine cervix from a pre-menopausal woman, undergoing a hysterectomy because of benign gynecological conditions. **B** Sagittal view of a cervix at gestational week 12 with a color box superimposed. The *anatomic area of interest* is marked with red. *Artifact in the anterior vaginal fornix

Therefore, the aim of this study was to provide recommendations for strain elastography of the uterine cervix by elucidating the crucial factors to improve this technique. These recommendations should be based on the literature and on the screening of homogeneous simulators with a known elastic modulus, as well as on pregnant women (Figs. 1, 2, 3).

## Materials and methods

### Terminology

**Fig. 4:**
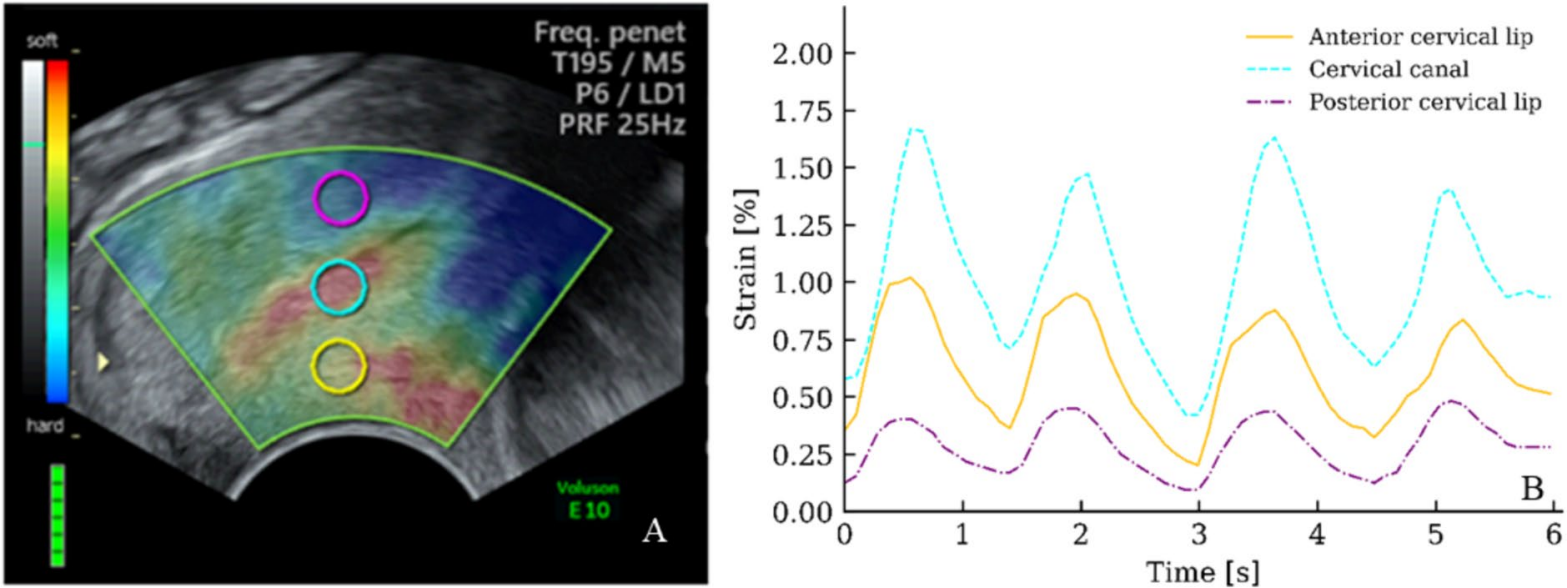
Influence of cervical canal **A** B-mode image with the color box superimposed onto a uterine cervix at gestational week 12. Three ROIs (⌀ 5 mm) are placed within the anterior cervical lip, in the cervical canal, and in the posterior cervical lip. **B** Distance-adjusted strain curves obtained from the three ROIs

**Fig. 5:**
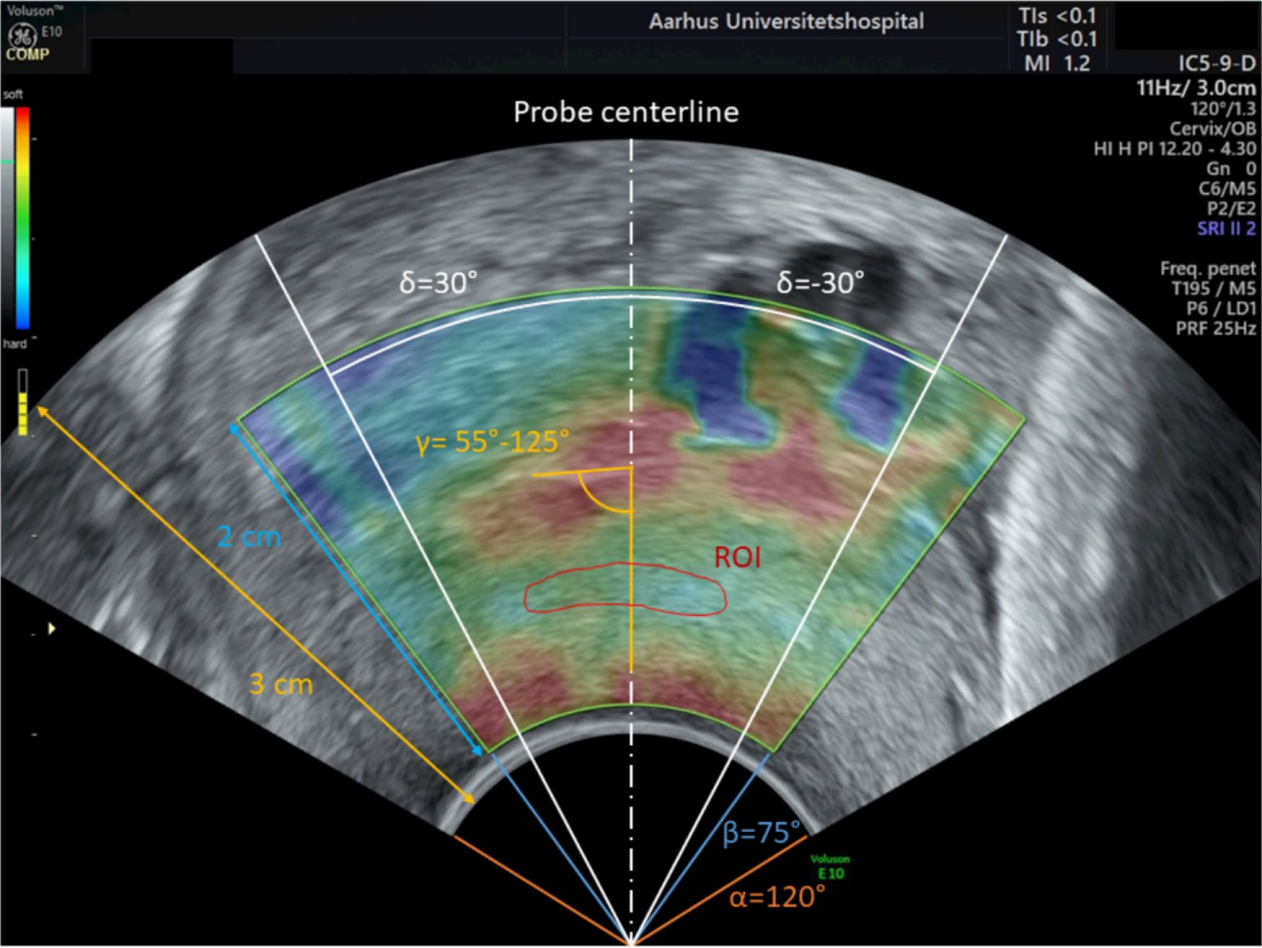
Proposed recommendations. B-mode image with a sagittal view of a cervix at gestational week 38^+0^ with a color box superimposed

- The *anatomic area of interest* refers to the cervical tissue that determines the biomechanical strength of the cervix (Fig. 4).
- The *probe-centerline* refers to the longitudinal axis of the transvaginal probe (Fig. 5).
- The c*olor box* is the cone-shaped box (Fig. 5) superimposed on the B-mode image when performing elastography scans.
- The *region of interest (ROI)* determines the tissue from which the strain values are computed (Fig. 5).
- The *computed strain values* within a chosen ROI were computed by the General Electric (GE) ultrasound system, using the cross-correlation technique.

### Ultrasound equipment and software

All scans were performed with a two-dimensional transvaginal ultrasound probe (IC5-9-D), connected to a Voluson^™^ E10 Expert scanner with the BT18 software, from GE Healthcare, Zipf, Austria.

At the ultrasound system, the option obstetric and cervix were chosen prior to all scans. The default settings used were the following: gain: 0; dynamic contrast: 6; magnification factor: 1.3; elasto map: 5; persistence filter: 6; line density: 1; window length: 22; window step: 4; filter axial: 2; filter lateral: 7; frame reject: 0; pixel reject: 0; transparency: 195; pulse repetition frequency: 25 Hz; and lateral resolution: 0.5 cm; smoothing filter: average 3 sample; frame rate: 10 Hz; frequency of the probe: 11 Hz.

### Scanning simulators

The simulators were made of polyacrylamide hydrogel (PAM) with an elastic modulus of 0.05 MPa and a total volume of 500 mL. The PAM was polymerized at 50 ℃ for 1.5 h by the use of 0.456 g potassium persulfate (PPS, 99%, SigmaAldrich); 3.424 g *N*,*N′*-methylenebisacrylamide (BIS) (95%, SigmaAldrich); 119.896 g acrylamide (AA) (95%, SigmaAldrich); 7.5 g Polystyrene (PS) nanoparticles and 1000 mL water (18MΩcm, MilliQ, UK). Oxygen was removed by flushing the solution with nitrogen prior to and during polymerization. Biomechanical testing of the simulators was performed on a universal Tensile Tester (Zwick/Roell GW7400.5a) using testXpert^®^ II V3.61 software. The elastic modulus was calculated from the stress/strain curve obtained by compression (i.e., ball indentation) of the PAM.

Simulators were scanned by placing the probe perpendicular to the surface. To assess the impact of the distance, three ROIs (⌀ 5 mm) were placed with their centers 5.3 mm (reference ROI), 11.5 mm and 17.5 mm from the probe (Fig. 1). Strain values were read off for all three ROIs from 75 image frames (approximately 7 s), and the mean ratios, including the standard deviations (SD), were computed.

To assess the influenced of the angle from the probe-centerline to the ROI, three ROIs (⌀ 5 mm) were placed 0° (reference ROI), 20° and 40° to the probe-centerline (Fig. 2). Strain values from 75 image frames (~ 7 s) were read off, and the mean ratios and SD were computed.

### Scanning pregnant women

The same operator (CRT) certified in cervical assessment by the Fetal Medicine Foundation scanned more than 75 women with gestational age between 12^+0^ and 41^+6^ weeks. They all had an empty bladder, and the probe was placed in the anterior vaginal fornix for a sagittal image of the uterine cervix, in accordance with the FMF recommendations [32].

To assess the impact of the cervical canal, we scanned 12-week pregnant women. Three ROIs (⌀ 5 mm) were placed within the anterior cervical lip, within the cervical canal, and within the posterior cervical lip, respectively. Strain values from 65 image frames (~ 6 s) were read off, adjusted for distance by the correlation factor obtained from the simulators, and the mean ratios and SD were computed.

### Review of the literature concerning the adherence to the present elastography recommendations

A literature search was conducted at the PubMed Database (February 16, 2024) using the MeSH Terms ‘elastography’ and ‘cervix uteri’. Only English language papers were included (*n* = 56). Two authors (NU and MSSJ) scrutinized the publications and based their judgements on the information concerning [1] the B-mode image, [2] the default settings, [3] the *anatomic area of interest*, [4] the ROI angle deviation, and [5] the ROI displacement.

### Ethical approval

Participants gave their written informed consent, and the study was conducted according to the Declaration of Helsinki and approved by the Danish Regional Committee on Health Research Ethics (1-10-72-138-16) and the Danish Data Protection Agency (2012-58-006). For histologic examination of the uterine cervix, a pre-menopausal woman undergoing a hysterectomy was included.

### Statistics

Figures are given as means and standard deviations (SD). We used polynomial regression to calculate a formula which normalizes the strain to a standard distance to the ultrasound probe.

## Results

### The anatomic area of interest within the anterior cervical lip

The elastogram illustrates a significant heterogeneity within the anterior cervical lip (Fig. 3B). According to the histologic preparation (Fig. 3A), the red and yellow areas (indicating very soft and soft tissue, respectively) at the elastogram along the cervical canal are associated with the peri-endothelial area next to the cervical canal as well as the sub-epithelial area below the cervical surface. Furthermore, a red area often appears beyond the exocervix (marked with * in Fig. 3B). This may indicate soft tissue, blood vessels (Fig. 3A), and smooth muscles [33] as well as artifacts probably caused by mucus in the anterior vaginal fornix. In both the histologic preparation and at the elastogram, the central third of the anterior cervical lip is more homogeneous. This area is dominated by a dense strength determining connective tissue [34] i.e., this area constitutes the *anatomic area of interest* regarding assessment of the biomechanical strength of the uterine cervix.

### Distance between ROI and probe

Due to signal attenuation, strain values from the simulator decreased with increasing distance from the probe to the ROI (Fig. 1). Compared to the strain of ROI_5.3 mm_—strain (ten recordings), the ROI_11.5 mm_—strain decreased by 30.7% (SD 5.1%) and the ROI_17.5 mm_—strain by 52.6% (SD 12.9%). To compensate for this signal loss, a mathematical signal amplification can be applied (Fig. 1C). Using polynomial regression, the correction function is given by *C* = 2.803⋅10^–3^ *x*^2^ + 2.675⋅10^–2^ *x* + 0.7737, where C is the amplified signal and *x* is the distance from the probe surface to the center of the ROI, measured in mm*.* This normalizes the strain to a distance of 5.3 mm from the probe surface.

### Angle between ROI and centerline

Strain values from the simulator decreased with increasing angle from the probe-centerline to the ROI (Fig. 2). Thus, when compared to ROI_0°_—strain (10 recordings), the ROI_20°_—strain decreased by 1.9% (SD 9.41%) and the ROI_40°_—strain by 59.4% (SD 19.0%).

To ensure proper compression of the *anatomic area of interest*, it is crucial that the compressions are along the probe-centerline, and the cervical canal is perpendicular to the probe-centerline. However, achieving this alignment can be challenging in clinical practice, particularly in early pregnant women. By allowing an angle of 90° ± 35°, i.e., an angle of 55–125° (*γ*), elastography scans can be conducted in most pregnant women still obtaining reliable results (Fig. 5).

### Cervical canal

Elastography studies on simulators have revealed that when a soft tissue in interposed between the probe and the ROI, there is a significant reduction of the obtained strain value [20]. This reduction falsely indicates that the tissue is harder than it actually is. Similarly, in the uterine cervix, the presence of the cervical canal induces a similar phenomenon (Fig. 4). Within the color box, the posterior cervical lip appeared bluer (indicating very hard tissue) compared to the greener (indicating hard tissue) anterior cervical lip. Consistent with this observation, the computed strain values, adjusted for distance, of the posterior cervical lip were 54% (SD 6.5%) lower than those obtained from the anterior lip (3 recordings).

### Elastography scans

It is important to keep the settings of both the B-mode scanning and the elastography option fixed as they affect the computed strain values [31]. Our elastography scans of both first trimester and term pregnant women showed that a B-mode image with a depth of 3 cm and an angle of 120° (*α*) ensures adequate cervical magnification for proper analysis, still maintaining an overview. A color box with a depth of 2 cm, an angle of 75° (*β*), and the middle centered at the probe-centerline was sufficient to include the *anatomic area of interest* (Fig. 5).

### Elastography analyses

Elastography analysis of scans obtained from pregnant women with different gestational ages showed that:

- To achieve a ROI as big as possible but within the *anatomic area of interest*, a ROI depicted by free hand is superior to a standardized circle-shaped ROI (Fig. 5).
- A small buffer zone around the ROI, but within the *anatomic area of interest,* is required in order to ensure that the ROI does not include tissue outside the *anatomic area of interest* during the compression–decompression cycles.

## Discussion

The findings highlight several of pitfalls that challenge cervical strain elastography. Consequently, we have elaborated recommendations regarding the settings of the elastography equipment, the conduct of the recording, and the position of the ROI (Table 1, Fig. 5).

**Table 1:** Recommendations for cervical elastography

1. Elastography
1.a Default settings
(a) The B-mode image
(i) Depth: 3 cm; angle, *α* = 120°
(b) The color box
(i) Depth: 2 cm; angle, *β* = 75°
(ii) The center of the box should be at the probe-centerline
(iii) The box should be placed as close to the probe as possible
(c) Default settings should not be modified either during or between scans
1.b Recording
(a) The B-mode image
(i) The probe-centerline should be perpendicular to the cervical canal though a deviation of 90° ± 35° is acceptable (*γ* = 55°–125°)
(b) Superimpose the color box
(i) The anatomic area of interest must be included in the box
(c) Compressions
(i) During the compression–decompression cycles, the deformation of the anatomic area of interest must be kept to a minimum, still ensuring a high quality elastography signal
(ii) Compressions and decompressions must be performed along the probe-centerline
2. Post-processing; elastography analysis—Region of Interest (ROI)
2.a Identifying the anatomic area of interest
(a) The recording should be browsed to identify the connective tissue in the middle third of the anterior cervical lip i.e., the anatomic area of interest. This area will normally appear more homogeneous and harder, according to the colors in the color box
2.b Placing the ROI
(a) A traced ROI is preferable to the standardized circle ROI
The ROI should
(i) Be placed within the anatomic area of interest
(ii) Be placed within δ = 30° from the probe-centerline
(iii) Not include the cervical canal; the soft tissue next to the cervical canal, or the outer surface of the cervix
(iv) Be surrounded by a small buffer zone, which ensures that the ROI remains within the anatomic area of interest during the compression–decompression cycles

The ROI must be placed within the *anatomic area of interest*, specifically the middle third of the anterior cervical lip. When scanning from the anterior fornix, computed strain values obtained from the posterior cervical lip are considered unreliable because of the interposed soft area within and along the cervical canal and because of the distance to the probe (Figs. 1, 3).

It is crucial to avoid the inclusion of irrelevant tissue within the ROI. This may occur if the force applied during elastography compression distorts the *anatomic area of interest*. Therefore, this force must be balanced to ensure an adequate elastography signal while minimizing the risk of distortion. In addition, when placing the ROI, it is important to leave a small buffer zone within the *anatomic area of interest,* even though the ROI should be as large as possible. Some ultrasound machines include software that may compensate for this [35].

The default settings of the ultrasound machine for elastography, such as the dimensions of the B-mode image and the color box, must not be adjusted during and between the scans, as this will render the strain values incomparable. When choosing the default settings, one should consider that a low framerate (e.g., below 20 Hz) may reduce the reproducibility of the strain assessments. It is important to note that the dimensions of the color box, in particular, influences this variable. Further, one must acknowledge that these settings may not be universally applicable to other strain elastography devises.

Many studies of cervical elastography utilize protocols that deviate from the recommendations outlined in Table 1. Selection the *anatomic area of interest* appears to be particularly problematic (Table 2). It is therefore hypothesized that adherence to the suggested recommendation could potentially strengthen the observed correlations between elastography recordings and the risk of preterm birth [9, 11, 27–29, 36–41].

**Table 2:**
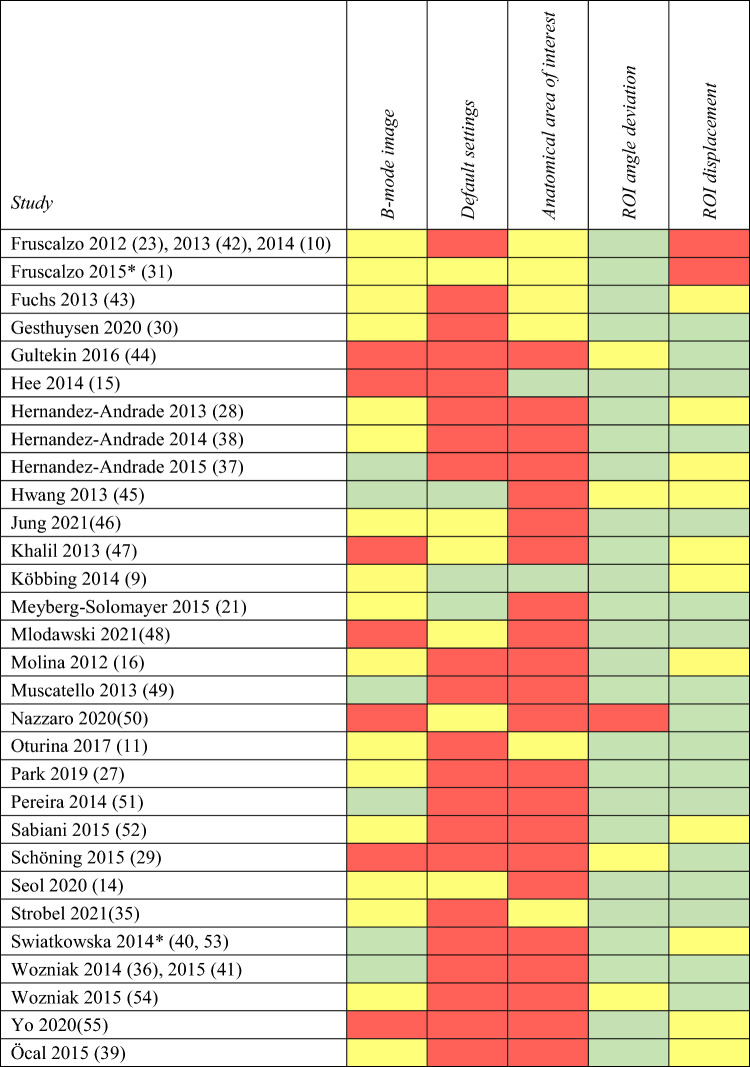
Overview of approaches used in publications on strain elastography on the human uterine cervix

B-mode image: the anterior cervical lip occupies GREEN > 40%; YELLOW 25–40%; RED < 25% or is not described/shown. Default settings: GREEN = completely described; YELLOW = partially described; RED = not described. Anatomic area of interest: GREEN = the mid cervical area only; YELLOW = the mid cervical area with the exocervical area and/or the endocervical area; RED = the entire cervix or the cervical canal or is not described/shown. ROI angle deviation (from probe-centerline): GREEN < 30°; YELLOW 30–45°; RED > 45° or is not described/shown. ROI displacement: During compressions, the ROI moves GREEN < 20%; YELLOW 20–50%; RED > 50% or is not described or shown. For this variable, we have permitted ourselves to assess if the ROI moves during compressions, if this was not described or shown

*Includes two articles from Swiatkowska-Freund from 2014. It is assumed that they used the same method.

Therefore, we propose that future studies on cervical strain elastography adhere to a standardized technique to enhance consistency and comparability of results. The recommendations outlined in this study can help mitigate common pitfalls and facilitate comparisons across studies. Moreover, advancements in equipment, such as incorporating a force-measuring device to gage compression force during examinations [19], implementing devices to ensure standardized compressions, utilizing reference material to be interposed between the cervical tissue and the probe, and developing automated strain value calculation software [14], are essential for further improvement in this field.

## Data Availability

The data will be available from the corresponding author on reasonable request.
